# Editorial: The key role of Mer receptor tyrosine kinase: where inflammation ends and fibrosis begins

**DOI:** 10.3389/fimmu.2023.1251577

**Published:** 2023-07-17

**Authors:** Gaetano Zizzo, Philip L. Cohen

**Affiliations:** ^1^ Temple Autoimmunity Center, Temple University, Philadelphia, PA, United States; ^2^ Unit of Rheumatology, Department of Internal Medicine, Azienda Socio-Sanitaria Territoriale (ASST) Ovest Milanese, Milan, Italy; ^3^ Section of Rheumatology, Department of Medicine, Lewis Katz School of Medicine at Temple University, Philadelphia, PA, United States

**Keywords:** Mer receptor tyrosine kinase (MerTK), CD206, CD163, macrophages, efferocytosis, cancer, fibrosis, arthritis

It has been nearly 30 years since the discovery by multiple research groups of the receptor tyrosine kinase family now generally referred to as the TAM (Tyro3, Axl, and MerTK) receptors (1). TAM kinases, particularly MerTK, are recognized to be of critical importance in efferocytosis, immune tolerance, and resolution of inflammation, as evidenced by tissue accumulation of apoptotic cells and development of systemic autoimmunity in mice lacking these receptors (2) as well as in humans with increased cleavage of TAM receptors into soluble ectodomains (3). They also appear important in promoting tumor growth (4) and tissue fibrosis (5–8), two challenging conditions often resistant to existing therapies. Interestingly, many of these functions are closely interlinked with the alternative activation of macrophages and differentiation of monocyte-macrophage progenitors into *M2c-like* cells (i.e., MerTK^hi^ CD206^hi^ CD163^hi^ CD16^hi^ CD14^+/hi^ CD204^+^ CD209^-^ Gas6^+^ IL-10^+^ pSTAT3^+^) (9–11).

The papers collected in this Frontiers Research Topic actually reflect the growing interest in exploiting the TAM receptor tyrosine kinases as novel therapeutic targets for tumoral, fibrotic, and immune-mediated diseases.

Jiménez-García et al. provide further insights into the key and ever more complex roles of MerTK and Axl in regulation of macrophage activities. Remarkably, mice lacking MerTK and Axl not only had a large increase in unengulfed apoptotic thymocytes, but also lower numbers of thymic and extrathymic macrophages and decreased expression of other functionally related scavenger receptor pathways, such as CD163 and CD206 (MRC1) (9), C1q (12), and Tim-4 (13), in the remaining phagocytes. Therefore, the expression of MerTK and TAM receptors in general is herein confirmed to profoundly affect homeostasis, survival, and polarization of macrophage populations. Of note, double-knockout mice also had dysregulated hemoglobin turnover, probably reflecting impaired CD163 expression, with iron accumulation in the kidney and anemia.

The tumor microenvironment, which is characterized by an overproduction of M2(c)-polarizing cytokines, such as M-CSF and IL-10, and an aberrant externalization of phosphatidylserine on tumor plasma membranes, may induce a strong upregulation of MerTK and its ligand Gas6 in tumor-associated macrophages (9, 14–16). The released Gas6 can in turn directly bind to TAM receptors on tumor cells, thus stimulating their survival and proliferation, or bind to MerTK on macrophages themselves, thus eliciting – in an autocrine/paracrine feed-forward loop – further production of IL-10 and immune tolerance to cancer, ultimately allowing for tumor cell migration and invasion (9). Therefore, novel strategies are being developed to block TAM kinases in cancer. Two articles in this series show the therapeutic potential of inhibiting MerTK pharmacologically or *via* targeted protein degradation. Cruz Cruz et al. tested the small-molecule MerTK inhibitor MRX2843 in a mouse model of acute myeloid leukemia, demonstrating its capability of repolarizing tumor-associated macrophages from non-inflammatory, tumor-permissive M2 (CD163^+^ Arg-1^+^ PD-L1^+^ PD-L2^+^ Tim-3^+^) to proinflammatory, anti-tumor M1 (CD86^+^ HLA-DR^+^), thus resulting in production of T-cell activating cytokines (IL-18, IFNβ) and conversion of infiltrating lymphocytes from exhausted/regulatory T cells (PD1^+^ Tim-3^+^ and LAG-3^+^) to activated/tumor-killing CTLs (CD8^+^ CD69^+^ CD107a^+^). The observed changes in both innate and adaptive immunity led to enhanced survival of leukemic mice, strongly encouraging the use of MerTK and TAM inhibitors, even in combination with other receptor tyrosine kinase inhibitors, immune checkpoint inhibitors, or chemotherapy, in the treatment of several neoplasms.

The work of Gadiyar et al. represents a novel and potentially powerful approach to modulating TAM receptors by using synthetic compounds that bind to one or more TAM kinases (including chimeric receptors) and also to the E3 ubiquitin ligase, thus leading to both inhibition of tyrosine kinases and degradation of protein complexes *via* the proteasome. These agents are shown to suppress MerTK expression and efferocytosis to levels approaching those of MerTK knockout mice, with more potent and prolonged action compared to small-molecule inhibitors, and possibly with no compensatory upregulatory mechanisms. On the other hand, some caution should be exercised regarding efforts to intensely downregulate or completely block the TAM receptors, in light of the diverse predicted and sometimes unexpected effects, as those reported by Jiménez-García et al. in double-knockout mice.

MerTK activation and M2(c) macrophage cross-talk with myofibroblast precursors, such as hepatic stellate cells in liver disease, have recently been implicated in fibrosis (7, 17). Here, Pipitone et al. highlight how MERTK gene polymorphisms can differentially influence the progression of chronic hepatitis C towards two predominant fates: advanced liver fibrosis or hepatocellular cancer. Specifically, the authors show that GG homozigosity in the rs4374383 SNP of MERTK is associated with higher expression levels of MerTK (and downstream AKT-interacting protein) and upregulation of WNT11 and SFRP1, which are involved in noncanonical WNT signaling leading to myofibroblast differentiation and fibrogenesis, whereas AA homozygosity is associated with decreased expression of MerTK and upregulation of metalloproteinases MMP7 and MMP9, which are instead involved in epithelial-mesenchymal transition and carcinogenesis. Therefore, detection of the AA (or AG) genotype in chronic hepatitis patients should imply closer surveillance against cancer, while therapeutic targeting of MerTK in patients with the GG genotype could be useful against cirrhosis.

Finally, an appreciation is emerging of the key role for MerTK^+^ pro-resolving M2(c) macrophages in fostering and maintaining remission of autoimmune rheumatic diseases, primarily rheumatoid arthritis (18–20). In this Research Topic, Royzman et al. studied a model of rheumatoid arthritis where mice received subcutaneous Freund’s adjuvant, followed by intraperitoneal *Bordetella Pertussis*, and finally intra-articular methylated bovine serum. Synovitis and bone erosions were reduced by administering a soluble form of CD83, which acted *via* the TLR4/MD2-TRIF pathway to inhibit IL-1β, IL-6, MMP9 and RANKL, while inducing MerTK, CD206 (MRC1), MARCO, and IL-10, thus resulting in a shift of macrophages from M1 (including osteoclast precursors) to M2. Of note in this context, engagement of CD14 would be crucial for soluble CD83 binding to MD2 (21), TLR4 endocytosis and TRIF activation (22), MerTK phosphorylation (23), and suppression of osteoclastogenesis.

Taken together, these studies help pave the way for new therapies aimed at inhibiting or facilitating the activity of MerTK and TAM-related pathways in different disease settings. We hope this Research Topic will stimulate further translational research focusing on these intriguing receptors.

## Author contributions

All authors listed have made a substantial, direct, and intellectual contribution to the work and approved it for publication.
